# Editorial: Mobile elements and plant genome evolution, comparative analyses and computational tools, volume II

**DOI:** 10.3389/fpls.2023.1308536

**Published:** 2023-11-10

**Authors:** Ruslan Kalendar, Gennady I. Karlov

**Affiliations:** ^1^ Institute of Biotechnology, Helsinki Institute of Life Science (HiLIFE), University of Helsinki, Helsinki, Finland; ^2^ National Laboratory Astana, Nazarbayev University, Astana, Kazakhstan; ^3^ All-Russia Research Institute of Agricultural Biotechnology, Russian Academy of Sciences, Moscow, Russia

**Keywords:** plant genome evolution, interspersed repetitive sequences, transposable element, retrotransposon, endogenous viral element, repeatome-based molecular markers

## Mobile element and host genome evolution

The genomes of eukaryotes are mostly composed of diverse families of interspersed repetitive sequences, including retrotransposons and transposable and endogenous viral elements. The prevailing view is that the diverse families of the genome repeatome should be considered only as parasites or “junk DNA” (Bourque et al., 2018). However, it is possible to follow genealogical trees, or pathways of evolutionary development and distribution of these elements, due to which, our understanding should be completely revised. The repeatome elements play a role that, in the sense of systems biology and medicine, goes far beyond “junk DNA” and viral fossils (Wells and Feschotte, 2020). Recent studies increasingly show that essential components, if not the most basic components of our genome, are of viral origin and that viruses as mobile genetic mediators have always played a crucial role in genetic evolution (Cosby et al., 2019). The evolution of genomes is associated with overcoming and fixing integrated events. With each important evolutionary step, the number of mobile genetic elements in the genome increased dramatically. Since the beginning of life, there has not been an organism that did not contain all these diverse mobile elements. In the formation of the genome, we can trace numerous processes involving mobile elements with their countless different appearances. Genomes are not the end product of innumerable accidental mutations and their selection, but a kind of living deposit from originally external, viral influences that is constantly being recycled and, like a chronicle, reinterpreted (Vassilieff et al., 2023). To be able to develop at all, mobile elements must have a coevolutionary relationship with their host genome (Gebrie, 2023). Evolutionary phylogenetic trees of mobile elements and the host genome show strong correlations (Kalendar et al., 2004; Kalendar et al., 2008; Moisy et al., 2014; Kalendar et al., 2020). Endogenous retroviruses, to which retrotransposons also belong, are single-stranded enveloped RNA viruses that are characterized by the fact that their genetic information, by means of reverse transcriptase, is rewritten into the DNA of the host genome and thus multiplies with each cell cycle (Johnson, 2019). If these retrotransposons enter the germline directly, they are not only passed on with each cell division but are also inherited and remain an integral part of the species genome. Retrotransposons inhabit almost all eukaryotic organisms without exception, and they can probably even be found as part of the genome of giant viruses. In most cases, retrotransposon-related elements live in the host genome and help the host resist infections and various forms of stress (Lanciano and Mirouze, 2018). Mobile genetic elements are crucial for species diversity and the evolution of the host genome. New genetic combinations always reveal the basis for new things for adaptive and developmental processes (Klein and Anderson, 2022). Diversity is always an indicator of vital and healthy ecosystems; this is true for the genome with its numerous families of interspersed repetitive elements. Thus, diverse families of repetitive elements are, genetically, one of the decisive factors in evolutionary innovation and species diversity.

We continued in the Research Topic “*Mobile Elements and Plant Genome Evolution, Comparative Analyses, and Computational Tools II*” to explore the effectiveness of new genomic tools to detect repetitive elements and highlighted some recent studies on the role of repetitive elements in host genome evolution, comparative analysis, and genome-wide profiling of retrotransposons and transposable and endogenous viral elements (Kalendar et al., 2021).

Plant genome evolution has mainly been determined by polyploidization and amplification or loss of retrotransposon-related elements. Research conducted by Mascagni et al. revealed that repetitive DNA within the *Olea* taxa constitutes a significant portion (ranging from 59% to 73%) of the total genome. This finding showcases substantial variations in terms of composition among these taxa. Notably, an intriguing observation emerged, namely the abundance of tandem repeats exhibited an inverse correlation with retrotransposons. For example, *Olea paniculata*, closest to *O. ancestor*, has few tandem repeats but abundant long terminal repeat retrotransposons, suggesting tandem repeat expansion post-divergence. This research unveiled the temporal dynamics that have played a pivotal role in shaping the genome structure throughout *Olea* speciation. This also provides a unique and insightful model for understanding the evolution of genomes in higher plants.

The genome of *Humulus scandens*, which is an important dioecious plant with XX/XY1Y2 chromosomes, was annotated with the repetitive portion of both the male and female genomes and compared with the different groups of repetitive sequences among the male and female genomes and with a close relative, *H. lupulus*. Zhang et al. analyzed the distribution of retrotransposons and satellite DNAs and determined the orientation position of the pseudoautosomal regions and indicated that the XX-XY1Y2 sex chromosomes of *H. scandens* might have originated from a centric fission event. Thus, this study revealed the nature of the origin and evolution of the sex chromosome of *H. scandens*.

## Discovery and comparative analysis of transposable elements

Endogenous viral elements (EVRs) are derived from DNA viruses of the family Caulimoviridae and abundant in plant genomes. de Tomás and Vicient analyzed 278 genome assemblies corresponding to 267 plant species to identify conserved domains of the reverse transcriptase of *Caulimoviridae*. These discovered EVRs were grouped in 57 clusters and classified in 13 genera, including a newly proposed genus *Wendovirus*. Comparing plant genomes, important differences between the plant families and genera in the number and type of endogenous pararetrovirus were found. In general, florendoviruses are the most abundant and widely distributed endogenous pararetrovirus.

The cold seasonal *Loliinae* subtribe includes taxa distributed worldwide and has a striking two-fold difference in genome size between the broad-leaved and fine-leaved *Loliinae* diploids and a general trend of genome reduction of some high polyploids. Moreno-Aguilar et al. used genome skimming data to uncover the composition, abundance, and potential phylogenetic signal of repetitive elements across 47 representatives of the main *Loliinae* lineages. The evolution of the *Loliinae* repeatome suggests a plausible scenario of recurrent allopolyploidizations followed by diploidizations that generated the large genome sizes of broad-leaved diploids and large genomic rearrangements in highly hybridogenous lineages that caused massive repeatome and genome contractions in the *Schedonorus* and *Aulaxyper* polyploids.

## Genome-wide profiling for transposable element analysis of repetitive elements

Interspersed repetitive elements are ideal for studying genetic variability in the genome and are crucial for studying the evolution of the host genome. Therefore, diverse high-throughput genotyping and sequencing applications have been developed. Arvas et al. described the main trends on promising directions of molecular marker technologies directly related to deployment of high-throughput genotype sequencing platforms.

The EG4 strain of rice is a unique material in that the transposon mPing has high transpositional activity and high copy numbers under natural conditions. Monden et al. identified the candidate genes and transposon mPing insertion sites that drive the high protein content of rice. The identified high-protein lines can lead to development of rice cultivars by introducing valuable traits, such as high and stable yield, disease resistance, and rich nutrient content.

Bread wheat genome evolution is largely dependent on a large number of diverse families of transposable elements (TE), which constitute approximately 80% of the genome. Bariah et al. found that about 36% of the 70 818 genes in bread wheat contained at least one TE insertion within the gene body, mostly in triads. TE insertions within the exon or in the untranslated regions of one or more of the homoeologs in a triad were significantly associated with homoeolog expression bias. A significant association was observed between the presence of TE insertions from specific superfamilies and the expression of genes associated with biotic and abiotic stress responses.

Ubi et al. studied 52 miniature inverted-repeat transposable element (MITE) insertion polymorphism markers for genetic studies in wheat and related species. Phylogenetic analysis of these MITEs insertions were consistent with the evolutionary history of these wheat species, which clustered mainly according to ploidy and genome types (SS, AA, DD, AABB, and AABBDD). The MITE insertion site polymorphisms uncovered in this study are very promising as high-potential evolutionary markers for genomic studies in wheat.

## Bioinformatic tools

To study and identify interspersed repetitive sequences and endogenous viral elements, specialized databases of these elements and bioinformatics tools for their *de novo* identification are needed.

A review of strategies used to identify transposition events in plant genomes is presented in a paper by Bajus et al. The authors described the basis of their operational principles to capture real cases of actively transposing elements and conceivable strategies. Combinations of methods resulting in improved performance are also proposed.

Argentin et al. performed a comparative analysis and classification of transposable element distribution in all plant species available in Ensembl plant genomes browser. The new classification of transposable elements was used to comparatively analyze the distribution of repetitive elements in 53 species (Wicker et al., 2007).

Mokhtar et al. developed the PlantLTRdb database containing retrotransposon sequences for 195 plant species. PlantLTRdb allows researchers to search, visualize, and analyze plant retrotransposons. PlantLTRdb can contribute to the understanding of structural variations, genome organization, functional genomics, and development of transposable elements targeting markers for molecular plant breeding.

## Conclusion

The prospects and challenges facing the exploration of repetitive DNA sequences and their role in genome evolution are both promising and intricate. Recent research has illuminated the pivotal role of repetitive elements in shaping evolution, driving genetic diversity, and regulating gene expression. However, the origins of transposable elements and their influence on genome evolution remain a significant puzzle in the realm of evolutionary biology. The co-evolutionary relationship between transposable elements and their host genomes stands as a key driver of genome size evolution, with the dynamics of this interplay potentially governing genome expansion and contraction. In-depth molecular studies have underscored the functional significance of repetitive elements, highlighting their necessity in orchestrating the expression of unique coding sequences and organizing essential functions crucial for genome operation. The repetitive genome component assumes a prominent architectural role in structuring higher-order genomic organization, while repetitive elements serve as invaluable tools for deciphering comparisons between sequenced genomes. The investigation of repetitive DNA sequences and their role in genome evolution is an intricate and ongoing discipline, offering tremendous potential for unravelling the origins and evolution of life on our planet.

## Author contributions

RK: Conceptualization, Funding acquisition, Investigation, Writing – original draft, Writing – review & editing. GK: Investigation, Resources, Validation, Writing – review & editing.
